# Strategies to Rescue the Consequences of Inducible Arginase-1 Deficiency in Mice

**DOI:** 10.1371/journal.pone.0125967

**Published:** 2015-05-04

**Authors:** Laurel L. Ballantyne, Yuan Yan Sin, Tim St. Amand, Joshua Si, Steven Goossens, Lieven Haenebalcke, Jody J. Haigh, Lianna Kyriakopoulou, Andreas Schulze, Colin D. Funk

**Affiliations:** 1 Department of Biomedical and Molecular Sciences, Queen’s University, Kingston, Ontario, Canada; 2 Vascular Cell Biology Unit, VIB Inflammation Research Center, Ghent, Belgium; 3 Department for Biomedical Molecular Biology, Ghent University, Ghent, Belgium; 4 Mammalian Functional Genetics Laboratory, Division of Blood Cancers, Australian Centre for Blood Diseases, Department of Clinical Haematology, Monash University and Alfred Health Centre, Melbourne, Australia; 5 Department of Paediatric Laboratory Medicine, The Hospital for Sick Children, Toronto, Ontario, Canada; 6 Division of Clinical & Metabolic Genetics, The Hospital for Sick Children, Toronto, Ontario, Canada; 7 Department of Paediatrics, University of Toronto, Toronto, Ontario, Canada; 8 Genetics and Genome Biology Program, Research Institute, The Hospital for Sick Children, Toronto, Ontario, Canada; University of North Carolina at Chapel Hill, UNITED STATES

## Abstract

Arginase-1 catalyzes the conversion of arginine to ornithine and urea, which is the final step of the urea cycle used to remove excess ammonia from the body. Arginase-1 deficiency leads to hyperargininemia in mice and man with severe lethal consequences in the former and progressive neurological impairment to varying degrees in the latter. In a tamoxifen-induced arginase-1 deficient mouse model, mice succumb to the enzyme deficiency within 2 weeks after inducing the knockout and retain <2 % enzyme in the liver. Standard clinical care regimens for arginase-1 deficiency (low-protein diet, the nitrogen-scavenging drug sodium phenylbutyrate, ornithine supplementation) either failed to extend lifespan (ornithine) or only minimally prolonged lifespan (maximum 8 days with low-protein diet and drug). A conditional, tamoxifen-inducible arginase-1 transgenic mouse strain expressing the enzyme from the *Rosa26* locus modestly extended lifespan of neonatal mice, but not that of 4-week old mice, when crossed to the inducible arginase-1 knockout mouse strain. Delivery of an arginase-1/enhanced green fluorescent fusion construct by adeno-associated viral delivery (rh10 serotype with a strong cytomegalovirus-chicken β-actin hybrid promoter) rescued about 30% of male mice with lifespan prolongation to at least 6 months, extensive hepatic expression and restoration of significant enzyme activity in liver. In contrast, a vector of the AAV8 serotype driven by the thyroxine-binding globulin promoter led to weaker liver expression and did not rescue arginase-1 deficient mice to any great extent. Since the induced arginase-1 deficient mouse model displays a much more severe phenotype when compared to human arginase-1 deficiency, these studies reveal that it may be feasible with gene therapy strategies to correct the various manifestations of the disorder and they provide optimism for future clinical studies.

## Introduction

In mammals, the urea cycle in liver metabolizes waste nitrogen. Urea cycle disorders (UCDs) in humans and corresponding mouse models have been described for each of the enzymatic steps in the pathway that is initiated by ammonia release from amino acids in hepatocytes [1]. The last step in the pathway, catalyzed by arginase-1, which converts arginine to urea, is essential for nitrogen disposal via the kidneys and for ornithine re-entry into the biochemical cycle [2].

Arginase-1 deficiency, also known as hyperargininemia, is an autosomal recessive disorder linked to more than 35 defined mutations spread at the chromosome 6q23 locus for *ARG1* [3,4]. Unlike the preponderance of UCD cases that present in the neonatal period with bouts of hyperammonemia and severe metabolic crisis, arginase-1 deficiency typically is discovered from late infancy to 2–4 years of age with signs that can resemble cerebral palsy [5]. These patients develop seizures, progressive spastic diplegia and learning disabilities that may become severe, along with the typical pattern of elevated plasma arginine [3–6]. A mouse model of arginase-1 deficiency was developed in 2002, but the mice died approximately two weeks after birth from apparent hyperammonemia [7]. These mice have been referred to as the “juvenile lethal” model of arginase deficiency [8]. To circumvent the early lethality of this model, inducible arginase-1 deficient mice were developed in order to study tissue- and temporal-dependent actions of arginase-1 [9–11].

Attempts to rescue the lethal consequences observed in the juvenile lethal model have been published [12–14]. Adenoviral transgene expression extended life by approximately 2 weeks [12], while adeno-associated viral (AAV) gene delivery of arginase-1 expressed from a strong promoter (chicken β-actin/CMV hybrid, known as CAG) rescued the lethality and allowed long-term survival, with some lingering biochemical and growth deficits but with apparently normal behavioral phenotype [13,14]. “Ectopic” transgene expression of arginase-1 in myocytes, rather than in hepatocytes, was able to partially reduce the elevated plasma arginine in the juvenile model but could not lengthen lifespan past 2 weeks of age [15].

Surprisingly, inducible arginase-1 deficient mice displayed many similarities to the juvenile lethal mouse model. Regardless of when the gene was knocked out from birth to adulthood (up to 12 weeks of age), the mice invariably died a few weeks later, with some differences between the two models that used either *Rosa26*-expressed Cre [10] or *Ubq*-expressed Cre [11]. Hyperargininemia, hyperammonemia and altered amino acid profiles were observed in the 2^nd^ week after inducing the knockout [10]. No studies have yet been presented on attempts to rescue the inducible knockout mice. Here, we present several strategies to rescue the mice and provide new insight into the strategies that do and do not work and what is required to correct inducible arginase-1 deficiency.

## Materials and Methods

### Induction of arginase deficiency in mice

**Fig 1:**
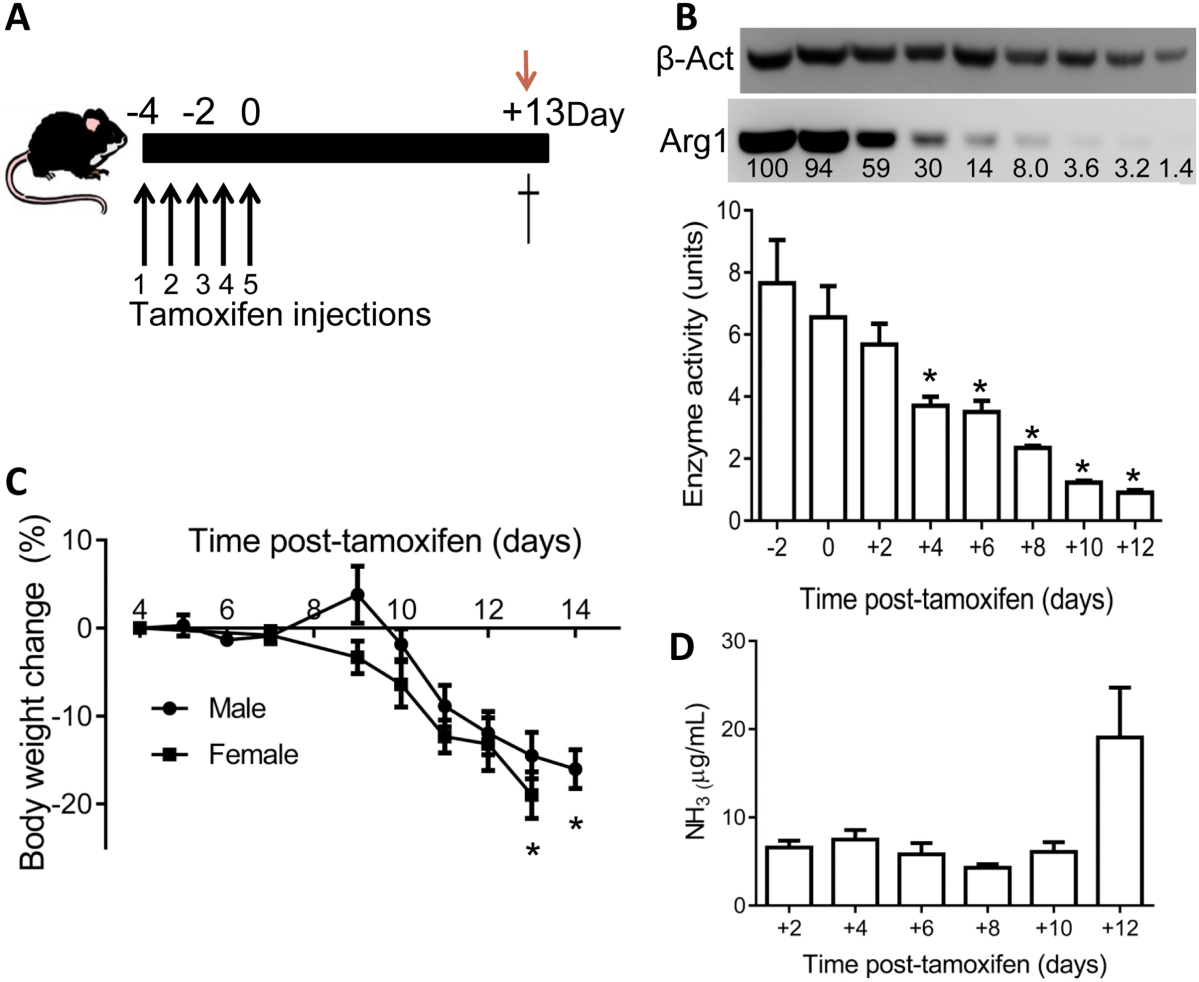
Floxed *Arg1-Cre* mice (8–12 weeks old) treated with the standard tamoxifen dosing regimen (A) show evidence of reduced liver arginase-1 enzyme activity and protein (n = 3 all points, except Day +12 where n = 4) over time (B). Day 0 is counted as the last day of tamoxifen administration, while Day +13 is the usual day for humane euthanization. Each Western blot lane (lower blot) corresponds to the enzyme activity bar below, with the exception that there is one extra lane in the blot corresponding to a mouse euthanized on Day +14. Image J quantitation relative to Day -2 is shown below the blot. The upper blot is the loading control for β-actin (β-Act). Enzyme activity was significantly decreased (*, *p*<0.05) compared to the “baseline” measurement (Day -2). (C) Typical phenotypic pattern of weight loss induced by Arg1 deficiency (n = 7 male; n = 5 female). Weight loss at humane endpoint was always significantly lower (*p*<0.05) than at Day +4. (D) Plasma ammonia in the same groups of mice used for protein and enzyme activity analysis. Day +12 levels were elevated but not significantly (*p* = 0.12).

Floxed *Arg1-Cre* mice of various ages and genders were injected daily for 5 consecutive days i.p. with tamoxifen, as we reported previously [10]. The timepoint designated as Day 0 for our studies is set as the 5^th^ tamoxifen dose, while the day of the initial dose is designated as Day -4 (see Fig 1A). PCR genotyping for the arginase-1 floxed (*Arg1* ^*fl*^) and Rosa26-Cre (*R26-Cre*) alleles was carried out as described [10]. All procedures were reviewed and approved by the Queen’s University Animal Care Committee (Funk 2011–048) and conformed to the Guidelines of the Canadian Council on Animal Care. Unless otherwise specified, water and standard rodent chow (containing 21.8% protein, 9% fat, 2.2% fiber, 5% minerals by weight; PicoLab Mouse Diet 20 (5058)) were provided *ad libitum*. Specific signs of health deterioration due to arginase deficiency were present in mice, regardless of gender, approximately 24 hours before exhibiting distress, which allowed for humane euthanization by CO_2_ inhalation [10]. Humane endpoints were defined as body weight loss of >15% relative to the weight at the time of the final tamoxifen administration, accompanied by hunched posture and poor grooming. Some mice could lose substantial weight in a single day when approaching the endpoint resulting in some exceeding the 15% threshold. This endpoint, in the absence of any type of “rescue” experimentation, took place without exception between Days +11 to +14 (n>200), with the mean at Day +13. Lifespan extension for subsequent experiments is in reference to this timepoint (+13).

### Biochemical analysis of plasma

Ammonia, guanidino compounds, arginine and various other amino acids were quantitated as previously described [10].

### Arginase activity assay

Arginase activities of all samples were assayed as described previously [10]. Briefly, liver tissues were homogenized in a solution containing T-PER protein extraction solution (Pierce) and 1× HALT protease inhibitor cocktail (Thermo Scientific) at 40 μl/mg tissue. Lysate (10 μl) was diluted with water to a final volume of 100 μl, followed by the addition of 100 μl of 25 mM Tris-HCl (pH 7.5) and 20 μl of 10 mM MnCl_2_. Samples were warmed to 56°C for 10 min followed by addition of 0.5 M L-arginine (pH 7.9) substrate (100 μl) and incubation at 37°C for 1 h. Arginase enzyme activity converts L-arginine to urea. The conversion was stopped by addition of acid mixture [900 μl of 1:3:7 mixture of H_2_SO_4_ (96%):H_3_PO_4_ (85%):water]. 1-phenyl-1,2-propanedione-2-oxime (ISPF) dissolved in ethanol was then added to each sample (40 μl of a 9% solution). The mixture was heated to 95°C for 60 min for development of a purple color for urea detection. Absorbance was measured at 540 nm (Varian Cary 50 Bio UV-visible spectrophotometer). Urea standards ranging from 0.02 to 0.5 μmol were used to create a standard curve for each assay. The extent of arginase enzyme activity was normalized to the protein content of each lysate, as measured by a Bradford reagent assay using standard protocols and 1 unit of activity corresponds to 10 nmol urea/μg protein.

### Western blot analysis

Liver tissues were homogenized in a solution containing T-PER solution and 1× HALT protease inhibitor cocktail (40 μl/mg tissue). Homogenates were further diluted in T-PER to a concentration of 1 mg/ml protein with 2x Laemmli buffer. Protein samples (20–30 μg) were subjected to Western Blot analysis. Thus, proteins separated by electrophoresis in 10% TGX FastCast acrylamide gels (Bio-Rad) were transferred to PVDF membrane (Bio-Rad, TurboBlot system) and probed with rabbit polyclonal anti-Arg1 (C-terminal peptide; 1:10,000 dilution; Abcam ab91279) and mouse polyclonal anti-β-actin (1:7500 dilution; Sigma) or anti-tubulin antibodies. Immunoreactive proteins were detected using HRP-conjugated goat anti-rabbit or anti-mouse secondary antibody (1:7500; Sigma) enhanced chemiluminescence signal. Digitized images were recorded with a FluorChem 8900 instrument (Alpha Innotech, San Leandro, CA). Some images underwent semi-quantitation with publicly available Image J software (Version 1.47, NIH).

### Ornithine supplementation study

A 1% ornithine (w/v; Sigma) solution was prepared in deionized water containing 5% sucrose and placed in regular bottles compatible with the vented cage racks. Control mice received only 5% sucrose in deionized water that was used to mask the unpleasant taste.

### Low-protein diet and nitrogen scavenging drug studies

Mice of different ages were switched from the normal chow diet containing 21.8% protein by weight to a diet with 6% protein (Harlan Laboratories,TD.90016), provided in pellet form *ad libitum*, starting 5 days prior to tamoxifen injections. Sodium phenylbutyrate was administered by daily oral gavage (100 μl of 100 mg/ml stock solution) to a cohort of mice (≈23 g body weight) on the low-protein diet.

### Generation and breeding of Arg1 transgenic rescue mice

A conditional *ROSA26* targeting vector construct encoding mouse arginase-1 was prepared by Gateway site-specific recombination methodology, essentially as described [16]. The construct was introduced into G4 (B6x129Sv/J) ES cells and clones were screened for evidence of homologous recombination. Five targeted ES cell lines tested positive by PCR for correct integration in the *R26* locus and for insertion of the Arg1 transgene (forward—AAAGTCGCTCTGAGTTGTTAT (*R26* allele; P1A); reverse—GGAGCGGGAGAAATGGATATG (*R26* allele; P1B); reverse—GCGAAGAGTTTGTCCTCAACC (splice acceptor insertion close to loxP site; P1C); forward—ATCATGTCTGGATCCCCATC (in 3rd pA sequence just before loxP site; P2A); and reverse-GGCGCTCCGATAATCTCTAA (*Arg1* allele (= P2B)). Two of these clones were expanded and chimeras were generated by aggregation with CD1 and/or Swiss Webster eight-cell stage embryos as described [16]. Subsequent breeding with C57BL/6 mice was carried out. One female mouse (born June 7, 2013) was found to have transmitted the genetic manipulation through the germline and was backcrossed again with a C57BL/6 male (N3) prior to mating with homozygous floxed *Arg1-R26 Cre* mice. Mice of 4 genotypes, either neonates or 4 week old, were treated with tamoxifen to induce Arg1 deficiency from the endogenous genetic locus [10] or to induce expression via the transgene.

### Quantitative real time PCR analysis of arginase-1 expression

Analysis of *Arg1* expression was carried out in liver, kidney and brain exactly as described previously [10].

### AAV construction and *in vivo* viral studies

Two types of recombinant AAV vectors encoding a murine Arg1-eGFP fusion (insert obtained from Origene MG204674 plasmid) were prepared. The first was driven by the liver-selective thyroxine-binding globulin (TBG) promoter with a chimeric intron from Promega (PI), encoding the 5′-donor site from the first intron of the human β-globin and the branch and 3′-acceptor site from the intron located between the leader and body of an immunoglobulin gene heavy chain variable region. It also contains the woodchuck hepatitis virus post-transcriptional regulatory element (WPRE) and bovine growth hormone (bGH) polyA signal sequence. The construct was packaged with viral capsids from AAV8 to yield AAV8.TBG.PI.Arg1-eGFP.WPRE.bGH with a titer of 3.06 x 10^13^ genome copies (gc)/ml.

The second construct was driven by a hybrid cytomegalovirus enhancer/chicken β-actin promoter (CB7), along with a chicken β-actin intron (CI), WPRE and the rabbit beta-globin (rBG) polyA signal. This construct was packaged with viral capsids from rh10 to generate AAVrh10.CB7.CI.Arg1-eGFP.WPRE.rBG with a titer of 1.82 × 10^13^ gc/ml. Vectors were produced by the Penn Vector Core at the University of Pennsylvania and information relating to the parent vectors can be obtained at: http://www.med.upenn.edu/gtp/vectorcore/. All vectors were purified by two rounds of cesium chloride gradient centrifugation, buffer-exchanged with PBS and concentrated with Amicon Ultra 15 centrifugal filter devices-100K (Millipore, Bedford, MA). Genome copy titer (gc/ml) was determined by real time quantitative PCR by the Core Labs as described [17]. Endotoxin assays (<5 endotoxin units per ml) passed specific release criteria by the Core Labs.

### Monitoring green fluorescent protein

The expression of GFP was examined by fluorescence microscopy of isolated hepatocytes and liver tissues. Primary hepatocytes were isolated from mice using a modified two-step collagenase perfusion system as described previously [10]. Liver sections were prepared from tissues by perfusion-fixation and/or immersion in 4% paraformaldehyde. Specimens were cryoprotected overnight in 30% sucrose, snap-frozen in Tissue-Tek OCT compound (Sakura, Torrance, CA, USA) and sectioned at 6 μm using a Leica CM1900 cryostat. Sections were then mounted with aqueous medium (Vectashield), visualized using a fluorescent microscope (Leica DM IRB, Richmond Hill, ON) and photomicrographs (x5, x10 objective) were recorded.

### Statistical analysis

All results are expressed as mean ± standard error of the mean (SEM). Statistical analysis was performed using Graph Pad Prism 6 (GraphPad Software, San Diego, CA, USA). Means were compared using the two-tailed Student’s t-test. *P* values of <0.05 were considered statistically significant.

## Results

### Temporal loss of arginase-1 expression and phenotypic changes in inducible arginase-1 knockout mice

To extend our previous studies with inducible arginase-1 deficiency, we sought to examine in more detail the loss of Arg1 protein and enzymatic activity in liver using our “standard” 5-day tamoxifen regimen (Fig 1A) that induces deletion of *Arg1* exons 7 and 8, as well as to observe phenotypic changes over the two week period prior to humane euthanization endpoint (Days +12 to +14 in the standard protocol). Very little change in Arg1 protein/enzyme activity is observed during the dosing schedule (Fig 1B) with ≈8 units enzyme activity at baseline and after 3 doses of tamoxifen (Day -2). However, by two days post-tamoxifen (Day +2) about 40% Arg1 protein is lost. Enzymatic activity is also lost but not in exact concordance with Arg1 protein, presumably due to some activity arising from the arginase-2 isoform, or other components in the liver homogenate that contribute to low level background readings in the assay. Previously, we had observed that biochemical changes (e.g. elevated arginine) begin to become apparent around Day +7. This is consistent with only 10% Arg1 protein and loss of about 40% enzyme activity at this time point. At endpoint (Day +13), there is consistently only 0.5–2% Arg 1 protein in all mice tested and less than 10% enzyme activity. Arginase enzyme activity levels in brain remain fairly steady regardless of time after tamoxifen administration and are less than 0.2% of normal levels found in liver (data not shown). The detection limit for the enzymatic colorimetric assay is presumably not sensitive enough to reveal tamoxifen-induced changes at this low level of enzyme activity and/or arginase-2 expression overrides that from arginase-1.

We estimate that once Arg1 protein/activity reaches a critical 10–15% threshold level there is an irreversible process set in motion (somewhere in the Day +7 to +10 stage with developing hyperargininemia) that culminates in the lethal phenotype, which is preceded by a very predictable weight and appetite loss that precedes the end-stage rise in plasma ammonia levels (Fig 1C and 1D and shown in subsequent sections). In fact, we have adopted weight loss, which is unrelated to tamoxifen administration [10], as a surrogate endpoint for health status/survival resulting from induced arginase-1 deficiency for all subsequent experiments.

### Ornithine supplementation elevates plasma ornithine levels but does not rescue the lethal phenotype

**Fig 2:**
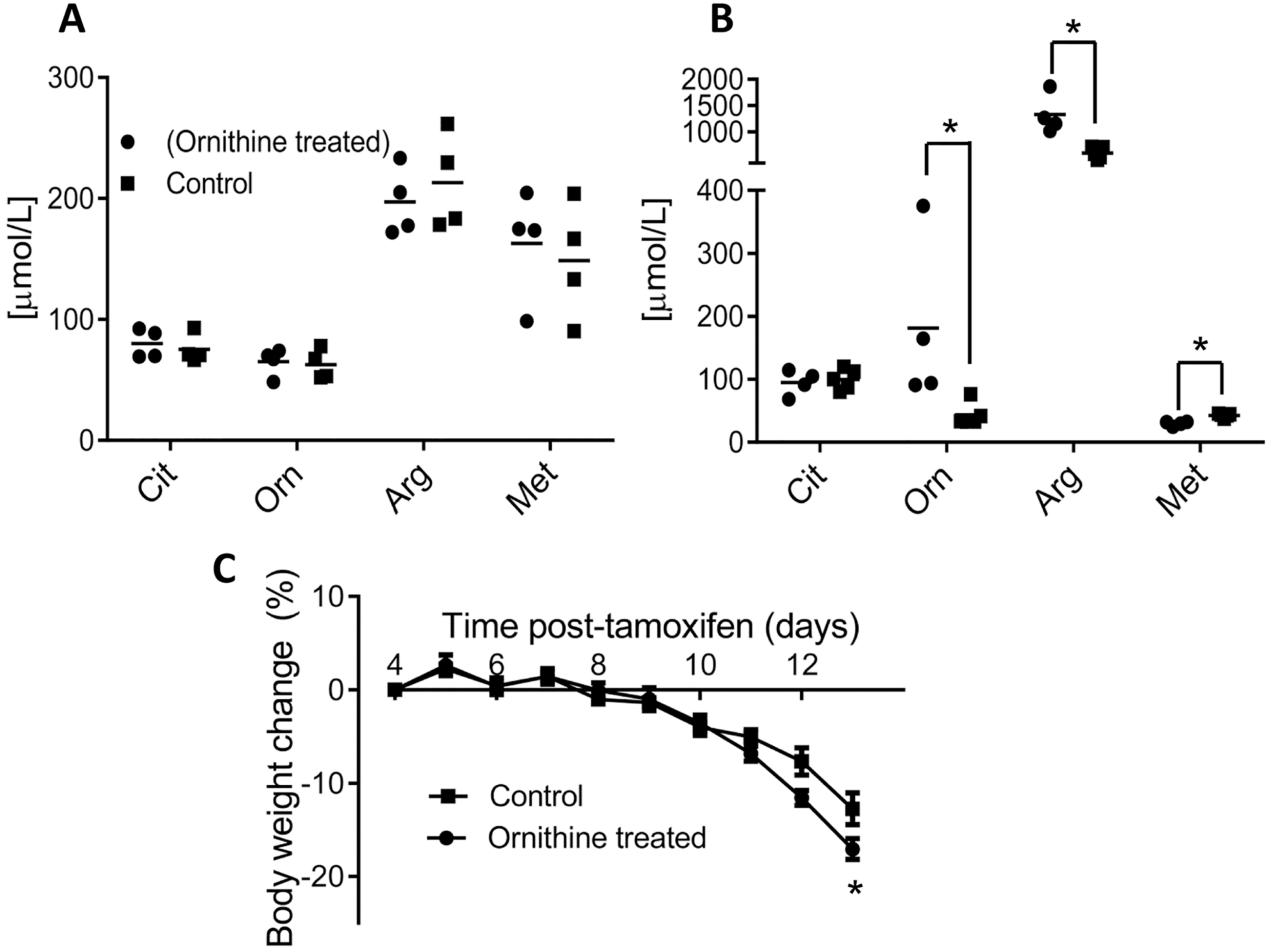
Ornithine supplementation does not rescue the induced arginase-1 deficiency phenotype. (A) Citrulline (Cit), ornithine (Orn), arginine (Arg) and methionine (Met) plasma concentrations are similar in control and ornithine-treated groups of 4 week old mice at baseline (n = 4). (B) Ornithine-treated mice (●, n = 4) indeed show elevations in plasma ornithine, which demonstrates the efficacy of the protocol, and have significant increases in arginine compared to control mice (■,n = 5) *, p<0.05 relative to control. (C) Mice in both groups show similar patterns of weight loss (surrogate marker of health status). Weight loss at humane endpoint was always significantly lower (*, *p*<0.05) than at Day +4.

We did not previously find any evidence for alterations in plasma ornithine in mice with induced Arg1 deficiency [10], as might be expected with the loss of this enzyme and as previously observed in the juvenile lethal model [7,8]. Low ornithine levels within liver hepatocytes, however, could be present as a result of diminished arginase activity and contribute to hyperammonemia and potentially to the lethality in the inducible Arg1 knockout mouse model. Therefore, we sought to supplement ornithine levels in a representative 4-week old cohort of mice by providing it in the drinking water. At baseline, all mice were healthy and there were no differences in amino acids, citrulline and ornithine between the groups of mice (Fig 2A and data not shown; n = 4). After tamoxifen dosing, hyperargininemia was present and ornithine was significantly elevated in the ornithine-supplemented group compared to controls (Fig 2B; n = 4 or 5). There was also a small, but significant, decrease in plasma methionine in the treated group with no alterations in any other amino acids including citrulline or glutamine. Tamoxifen treatment to induce Arg1 deficiency provoked the typical time course of weight loss (Fig 2C) and phenotypic decline resulting in humane endpoints at the usual time point in both groups of mice.

### Low-protein diet with or without sodium phenylbutyrate minimally extends lifespan of induced arginase-1 deficient mice

**Fig 3:**
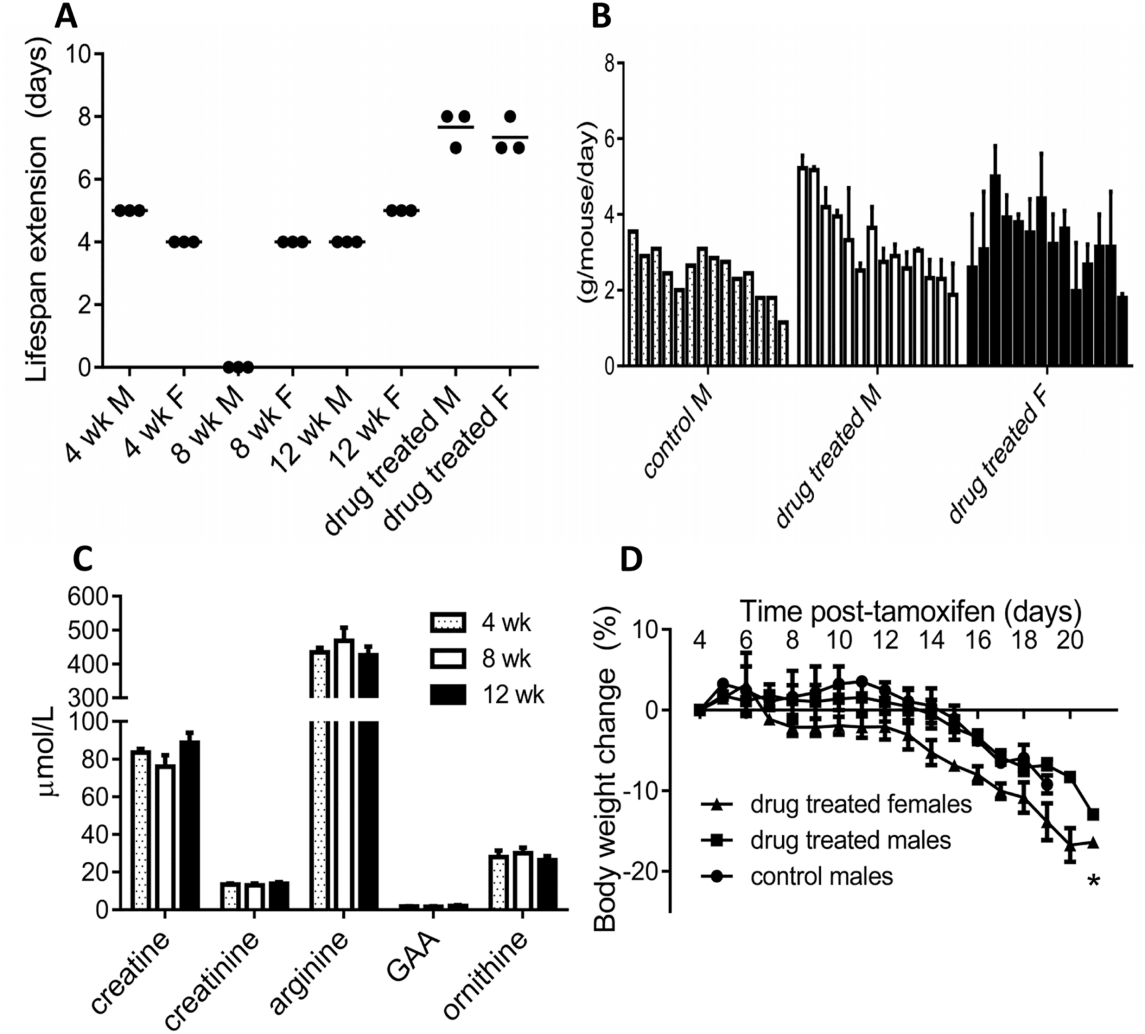
A low-protein diet, with or without sodium phenylbutyrate (drug treated), minimally extends lifespan of mice with induced arginase-1 deficiency. (A) M = male (n = 12, divided into four groups of 3), F = female (n = 12, divided into four groups of 3). (B) Food consumption on a low-protein diet with sodium phenylbutyrate (drug treated) or without (control, non-drug treated) tends to wane prior to humane euthanization. Each bar within a group represents an individual day from Day +4 (left side) to Day +17 (right side). (C) Plasma levels of five metabolites at endpoint in three groups of non-drug treated mice on a low-protein diet. (D) Typical body weight loss is observed in same mice used in (B). Weight loss at humane endpoint was significantly lower (*, *p*<0.05) than at Day +4.

Urea cycle disorder patients are maintained on a low-protein diet to reduce arginine, sometimes combined with ammonia-scavenging drugs, to limit potential hyperammonemic episodes [18]. 4, 8, and 12-week old male and female mice (n = 9 each gender) were maintained on a low-protein (6%) diet. Using the surrogate weight loss parameter, the time to humane endpoint was extended by 4–5 days in female mice of all three age groups and also in 4- and 12-week age groups of male mice (Fig 3A). Food consumption tapered off before endpoint in all groups consistent with a loss of appetite that we had previously observed [10] in mice eating a regular chow diet (21.8% protein; Fig 3B and data not shown). Plasma levels of arginine, creatine, creatinine, guanidino acetic acid and ornithine were measured, along with ammonia. Ammonia and arginine levels were elevated in all groups at endpoint compared to baseline control mice (Fig 3C and data not shown; baseline values in ref.10). The addition of daily sodium phenylbutyrate, administered by oral gavage, modestly extended lifespan of induced arginase-1 deficient animals by 3–4 days compared to low-protein diet alone, along with the diminishing pattern of food consumption and typical weight loss (Fig 3A,3B and 3D).

### Inducible transgenic arginase-1 expression from the *Rosa26* locus partially rescues knocked-out arginase-1 deficiency in neonates but not in 4 week old mice

**Fig 4:**
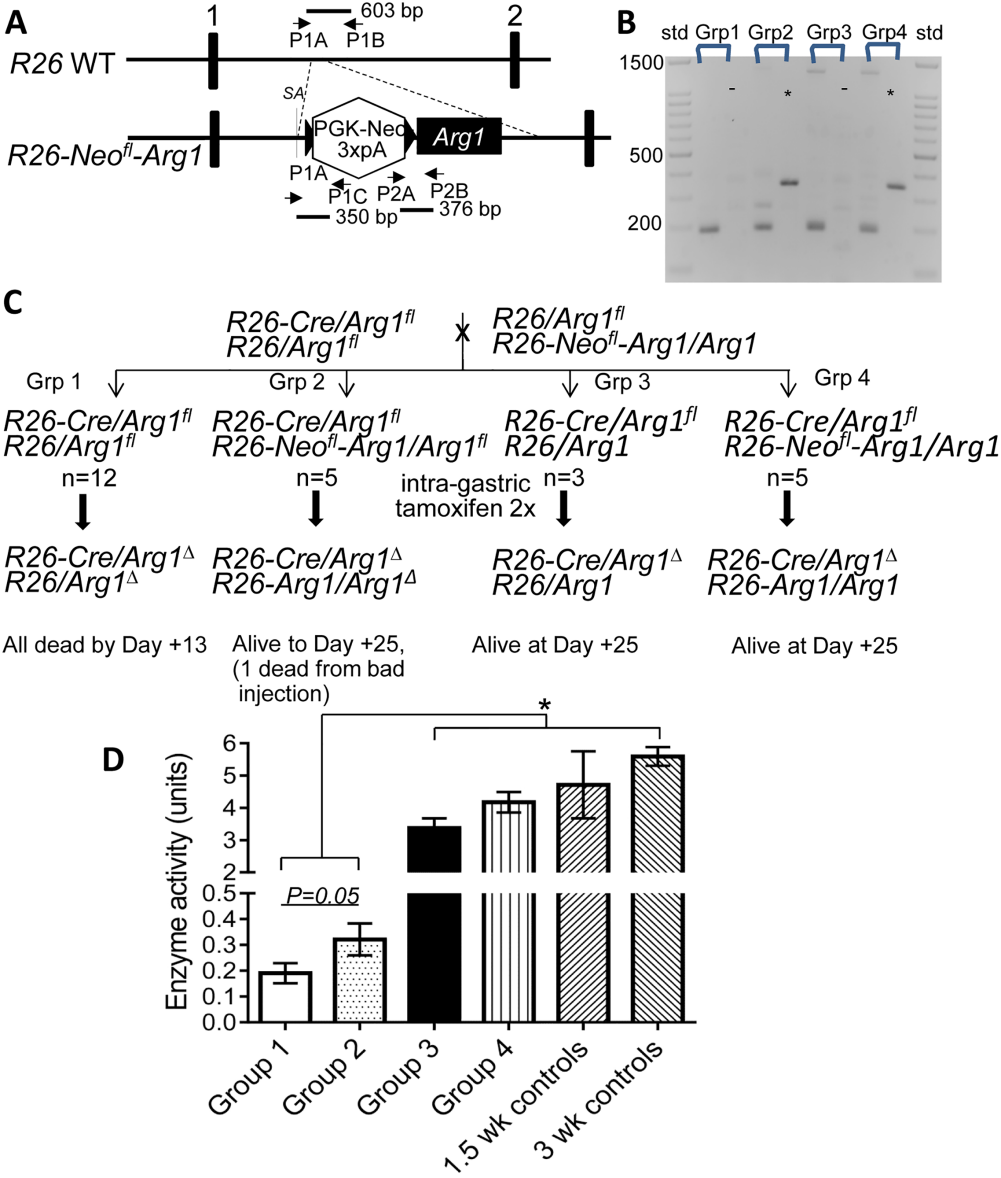
Transgenic mouse rescue strategy for Arg1 inducible knockout mice. (A) Generation of conditional *Rosa 26 (R26)*-promoter-based expression of Arg1, based upon ref [16]. Various primer sets used for genotyping and PCR products generated are shown. Exons (shaded rectangles), loxP sites (shaded triangles), selection cassette (hexagon) and transgene (shaded box) are depicted. SA, splice acceptor. (B,C) Genotypes of germline-transmitted cross-bred mice at *R26* and *Arg1* loci used to test inducible Arg1 transgene rescue of induced knockout of endogenous Arg1. Neonatal mice of 4 genotypes (n = 25, divided into groups as indicated) were administered tamoxifen on the day of birth and one day later, intragastrically, to generate the various Cre-excised alleles. (B) Representative PCR genotyping of each group (Grp). *, *R26-Arg1* transgene positive;-, *R26-Arg1* transgene negative with primers P2A/P2B, (R lane of each group). 195 bp band is *Arg1* ^Δ^ exon 7/8 Cre-excised allele, 252 bp band is residual *Arg1* ^*fl*^ allele after tamoxifen, and 1.2 kb band from *Arg1* wild-type allele (using primers A,B,C described in ref. 10; left lane each group). (C) Mice carrying different allelic combinations at the *R26* (left of /) and *Arg1* (right of /) genetic loci were cross-bred to yield mice of various genotypes (only 4 shown and studied). One mouse from Group 2 died from gastric injury at Day +8 as observed on autopsy examination. (D) Liver Arg1 enzyme activity (n = 3–4) at Day +13 (Group 1) or Day +25 (Groups 2–4), along with some C57BL/6 control mice of similar ages; *, *p*<0.05.

We devised a transgenic mouse “rescue” strategy whereby we could concomitantly elevate arginase-1 transgene expression ubiquitously while knocking out endogenous arginase-1. To do this, the mouse arginase-1 coding sequence was placed in the context of the well-known *Rosa26* (*R26*) gene intron 1 “safe-harbor” using embryonic stem cell gene targeting (Fig 4A), preceded by a “floxed” PGK-Neo resistance cassette [16]. Cre-mediated removal of the antibiotic resistance cassette should induce arginase-1 expression under the control of the *R26* promoter to drive widespread tissue expression. These mice, with no obvious overt phenotype, were crossed over a few generations with floxed *Arg1* (*Arg1* ^*fl*^) mice to generate mice with various allelic combinations at the *R26* and *Arg1* loci (e.g. *R26* expressing Cre^ERT2^ [19](*R26-Cre*) at one allele, the other expressing the floxed PGK-neo/arginase-1 transgene (*R26-Neo* ^*fl*^ *-Arg1*).

Four groups of neonatal mice were studied in the first experiment using intragastric tamoxifen administration to induce expression from the transgene and to knockout endogenous Arg1 (*Arg1* ^Δ^) in a coordinated manner (see Fig 4B and 4C). The two groups of mice (Groups 3 and 4) that expressed one normal *Arg1* allele all survived to day 25 and were healthy with no overt phenotype, at which point they were euthanized. All Group 1 mice expressing two *Arg1* ^Δ^ knockout (KO) alleles with no *Arg1* transgene died by Day +13, while Group 2 mice expressing two *Arg1* ^Δ^ KO alleles with the *R26-Arg1* transgene (TG) had lifespan extended to approximately 25 days of age (with the exception of one mouse that died from an inerrant intragastric tamoxifen injection). However, these mice eventually succumbed to the lethal consequences of Arg1 deficiency with correspondingly reduced Arg1 enzyme activity, elevated arginine and wasting (Fig 4D and data not shown).

**Fig 5:**
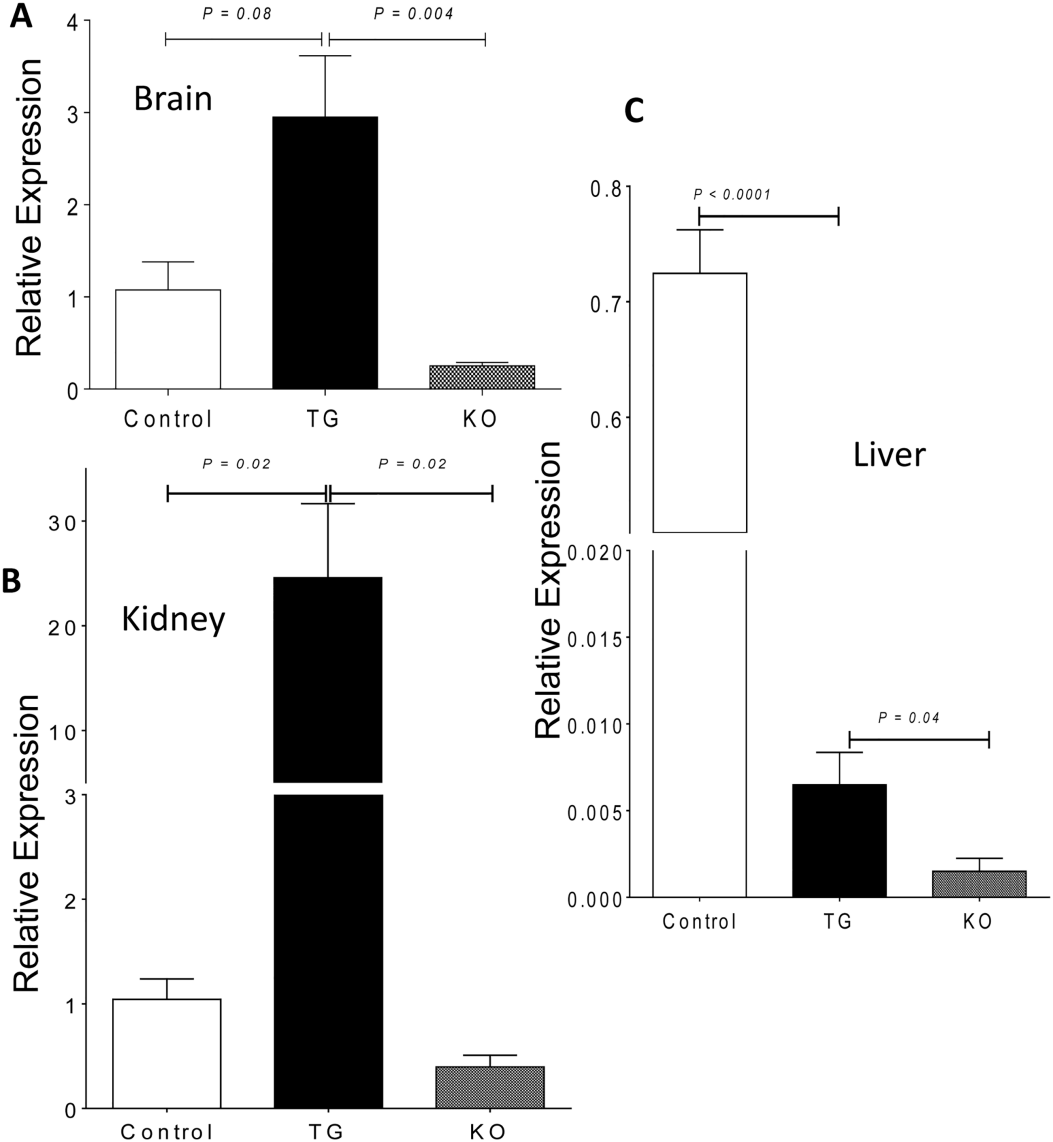
Real time quantitative PCR expression of *Arg1*. Brain (A), kidney (B), and liver (C) of control C57BL/6 (n = 3), *R26-Arg1* transgenic (TG; n = 3–5), and *Arg1* ^Δ^ knockout (KO; n = 3–5) mice.

**Fig 6:**
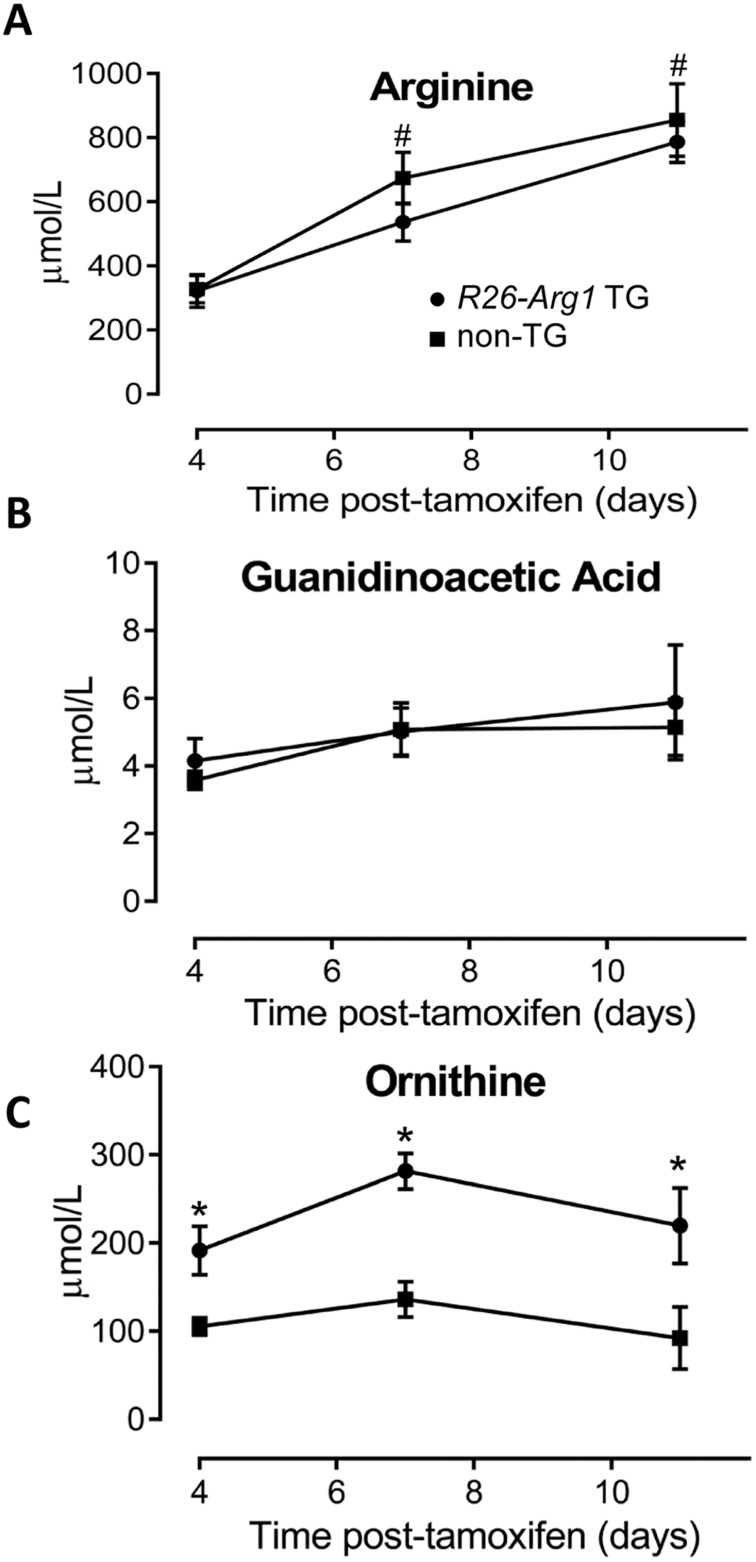
Measurement of plasma arginine (A), guanidinoacetic acid (B), and ornithine in tamoxifen-treated *R26-Arg1* transgenic (TG) and non-transgenic mice both on the *Arg1* KO background at three timepoints. #, p<0.05 when compared to Day +4. Ornithine levels differed significantly between groups (n = 5–7; *, p<0.05).

Since neonatal mice only showed signs of limited rescue, we next focused on 4 week old mice (control C57BL/6, *R26-Arg1* TG (Group 2), and *Arg1* ^Δ^ KO (Group 1) mice and sought to examine the relative change in *Arg1* expression due to the induced *Arg1* transgene in three tissues by real-time quantitative PCR. *Arg1* expression was significantly greater in liver, brain, and kidney of tamoxifen-treated *R26-Arg1* TG mice compared to *Arg1* ^Δ^ KO mice and was also greater than in control, non-tamoxifen treated C57BL/6 mouse brain and kidney (Fig 5). However, expression of *Arg1* in liver was substantially greater in control mice compared to the TG mice and this may explain why the *R26-Arg1* TG mice still continued to show signs of demise and were not rescued to any great extent at this age with hyperargininemia and modestly elevated guanidino acetic acid (Fig 6A and 6B). Plasma ornithine levels were elevated in the tamoxifen-treated TG mice compared to KO mice (Fig 6C).

### AAV-directed expression of an arginase-1-eGFP construct rescues some male mice

**Fig 7:**
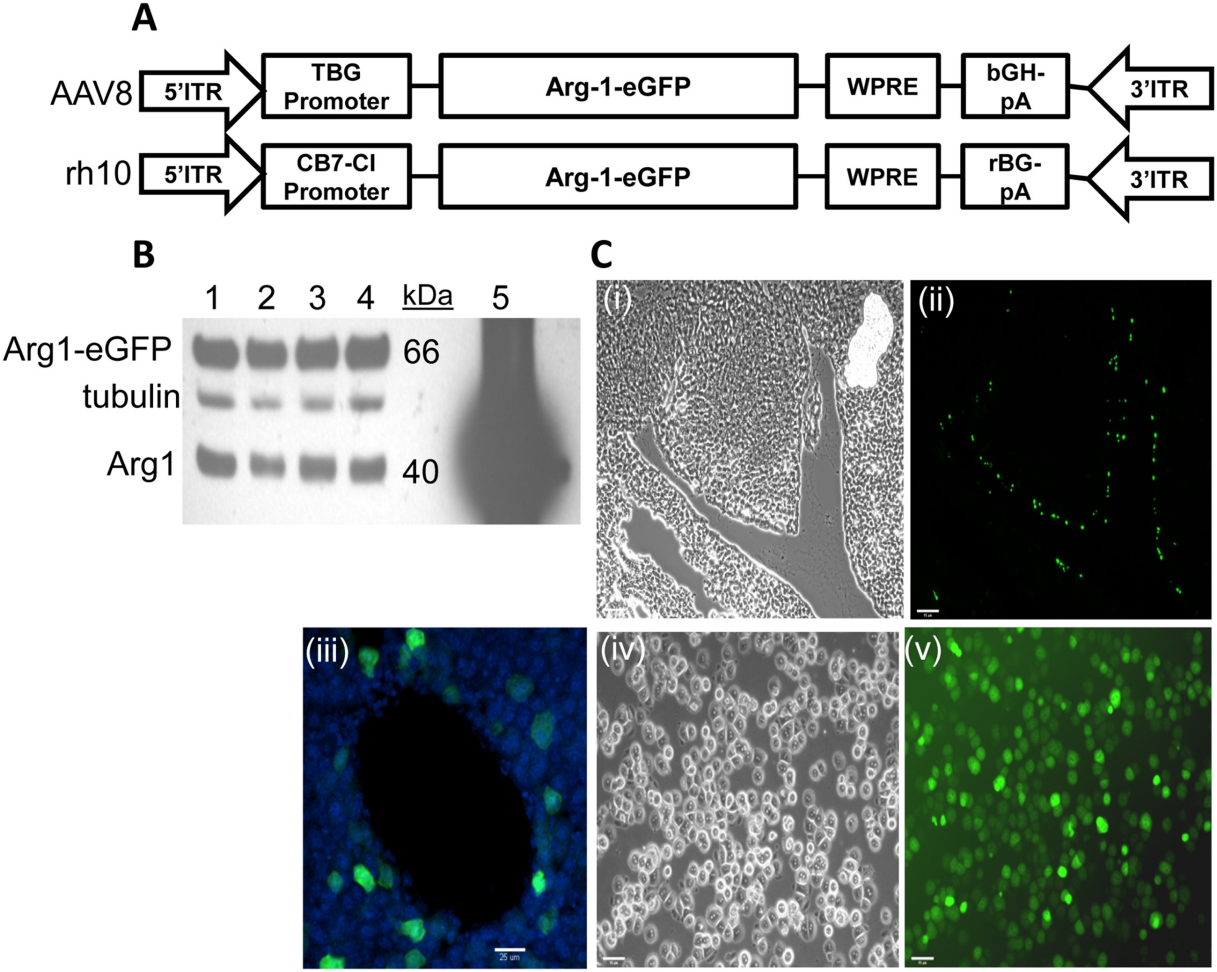
Generation of adeno-associated viral (AAV) vectors used in studies to rescue induced Arg1 deficiency. (A) Maps (not to scale) of two AAV constructs used and the packaging serotypes (AAV8 and rh10). ITR, inverted terminal repeat; TBG, thyroxine-binding globulin; CB7-CI, hybrid cytomegalovirus enhancer/chicken β-actin promoter (CB7), along with a chicken β-actin intron (CI); WPRE, woodchuck hepatitis virus post-transcriptional regulatory element; bGH-pA, bovine growth hormone polyA adenylation signal sequence; rBG, rabbit beta-globin polyA adenylation signal sequence. (B) Western blot indicating level of expression in livers of 4 separate mice (lanes 1–4) of AAV-expressed Arg1-eGFP relative to WT Arg1 and tubulin loading control post-tamoxifen (Day +13;4-week old mice injected with 2x10^*11*^ gc of the AAV8 construct shown in panel A) on Day -4. Lane 5 represents normal level of expression of Arg1 (and tubulin) in a mouse not injected with tamoxifen. In order to observe sufficient signal of the inducible Arg1 knockout band of lanes 1–4, the signal becomes over-saturated when detecting normal endogenous levels of Arg1 (lane 5). (C) Images of liver sections (i-iii) and dissociated hepatocytes (iv, v) from AAV8.TBG.PI.Arg1-eGFP.WPRE.bGH injected 4-week old mice 1 week after virus administration. Strong fluorescence signal from the fusion transgene is observed in the cytoplasm of some periportal hepatocytes (ii,iii) and also in dissociated cells (v) with corresponding phase contrast images (i, iv).

We first tried to rescue induced Arg1 deficiency using an AAV vector construct with primarily liver-specificity (TBG promoter and AAV8 serotype), encoding an Arg1-eGFP fusion protein (Fig 7A) in tamoxifen-treated, floxed Arg1-Cre mice that would allow for easy tracking of the protein in liver by fluorescence imaging. We started with small groups of mice (n = 3–4) varying three separate parameters: (i) the route of vector administration (i.v. vs i.p.); (ii) the viral dose (5x10^10^, 10^11^, 2x10^11^ gc/mouse); and (iii) the timing of viral delivery relative to tamoxifen-induced *Arg1* deletion (several timepoints from 5 weeks before (Day -35) to one week after (Day +7)) until 73 total mice were tested with this construct (Table 1). In general, even though a ≈66 kDa Arg1-eGFP protein expression could be detected by Western blot analysis and be distinguished from endogenous residual Arg1 protein (≈40 kDa; Fig 7B) and fluorescing cells were present in the liver in perivascular loci (Fig 7C(ii,iii)) and in isolated hepatocytes (Fig 7C(v)), almost all mice died in the usual timeframe (Table 1). We estimated that liver expression of the virally-delivered Arg1-eGFP never exceeded 3% of endogenous Arg1 levels based on the 1.3–1.7x greater densitometric signal of the transgene relative to the residual native Arg1 and an estimate of 1.5% residual native Arg1 after tamoxifen treatement (Fig 7B; cf. Fig 1B). We surmised that this level of expression was still too low to rescue mice from the lethal consequences of Arg1 deficiency. It should be noted that when native mouse Arg1 or Arg1-eGFP-encoded plasmids were transfected into HEK293 cells (prior to any viral constructs being generated), Arg1-eGFP was found to retain almost full catalytic activity compared to Arg1 (data not shown).

**Table 1:** Experiments to test AAV rescue of inducible arginase-1 deficient mice.

Expt # Date (mm/yy)	* Age (weeks)	n ( ♂ / ♀ )	Viral g.c.,delivery route, promoter/serotype	* Timing of viral administration	Comments (Mouse designation)
1) 05/13	4	25 (12/13)	2x10^11^; i.v.;AAV8/TBG	-9, -4, 0, +4, +7	No effect on lifespan
2) 07/13	8	36 (22/14)	10^11^ or 2x10^11^ i.v.; AAV8/TBG	-14, -21, -28, -35	3♂lifespan extended 5–6 days for Day -21 viral administration
3) 12/13	8	12 (7/5)	5x10^10^; i.p.;AAV8/TBG	-14, -21, -28, -35	No effect on lifespan
4) 05/14	12	11 (11/0)	5x10^10^ (n = 8) or 2.5x10^10^ (n = 3) i.p.; rh10/CB7	-3	4 mice lifespan extended by 7–8 days; others extended 2–3 days
5) 06/14	12	12 (5/7)	7.5x10^10^	-8	^#^ ♂tested at +21 (A2) ^!^
			10^11^		♂died
			1.5x10^11^		♂died at +17
			2x10^11^		2♂survived >25 weeks (A1, A10)
			i.p. rh10/CB7		All ♀ died at Day +17
6) 09/14	12	5 (2/3)	1.5x10^11^; i.p.rh10//CB7	-17, -31	2♂survived >12 weeks (S1, S4)
7) 11/14	12	6 (6/0)	7.5x10^10^	-17	For each viral dose, 1♂died at +14 (S7, S10) 1 at +18 (S8, S12), 1 survived >7 weeks (S9, S11)
			1.5x10^11^		
			i.p. rh10/CB7		

Notes:

*Age of mice is based on the day of the first tamoxifen administration.

Timing of viral administration is relative to the last dose of tamoxifen (designated 0) and is given in days (-14 refers to two weeks prior to the last dose of tamoxifen, while +7 refers to one week after the last dose. Several extra vehicle-injected control mice were included in the studies (not shown in Table) to exclude viral-mediated toxicity.

**Fig 8:**
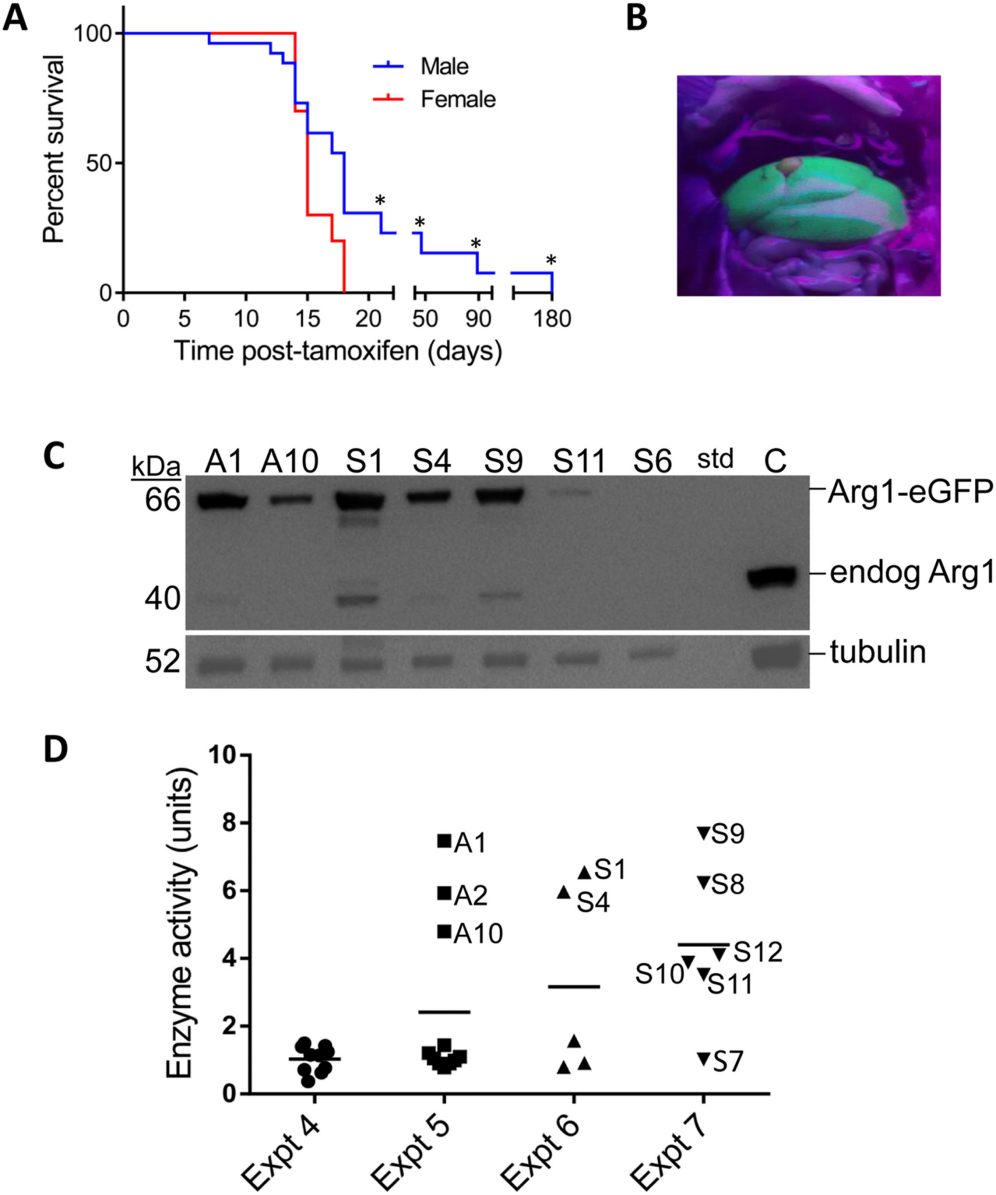
Some male mice are rescued from the lethal consequences of induced Arg1 deletion by AAVrh10.CB7.CI.Arg1-eGFP.WPRE.rBG administration. (A) Survival of tamoxifen-treated male (n = 24) and female (n = 10) induced Arg1 KO mice injected with AAV vector (Expts 4–7, Table 1). Seven “rescued” healthy mice were sacrificed at Day +21 (mouse A2, n = 1), Day +49 (mice S9, S11; n = 2), Day +90 (mice S1, S4; n = 2), and Day +180 (mice A1, A10; n = 2) for analysis of Arg1-eGFP expression. This explains the drop off in male survival beyond Day +21 denoted by asterisks. (B) *In situ* liver showing extensive green fluorescence expression from the Arg1-eGFP transgene in one rescued male mouse S1 sacrificed at Day +90, viewed under a handheld UV light (purple background). Note absence of expression in the gall bladder. (C) Western blot depicting Arg1-eGFP transgene expression in six of the rescued male mice mentioned above (mouse A2 not tested). The lane corresponding to mouse S6 was non-AAV-treated (Arg activity = 0.24 units; not shown in panel D). Protein standards (std) are not visible on the Western blot. Con, control non-tamoxifen treated mouse injected with an AAV-GFP construct. (D) Liver arginase enzyme activity at endpoint in AAV-injected mice of Expts 4–7 (Table 1). Activity of rescued (A1, A2, A10, S1, S4, S9, S11) mice are labeled, along with three mice not rescued but with substantial enzyme activity (S8, S10, S12).

^#^Mouse A2 was euthanized at Day +21, even though healthy (within 2% of starting body weight and expressing 75% of control liver arginase activity; see Fig 8D.

^!^Mouse died due to non-viral mediated testicular morbidity.

CB7, hybrid cytomegalovirus enhancer/chicken β-actin promoter; TBG, thyroxine-binding globulin promoter. Mouse designation corresponds with data found in Fig 8C and 8D.

^♂^, male;

^♀^, female.

**Fig 9:**
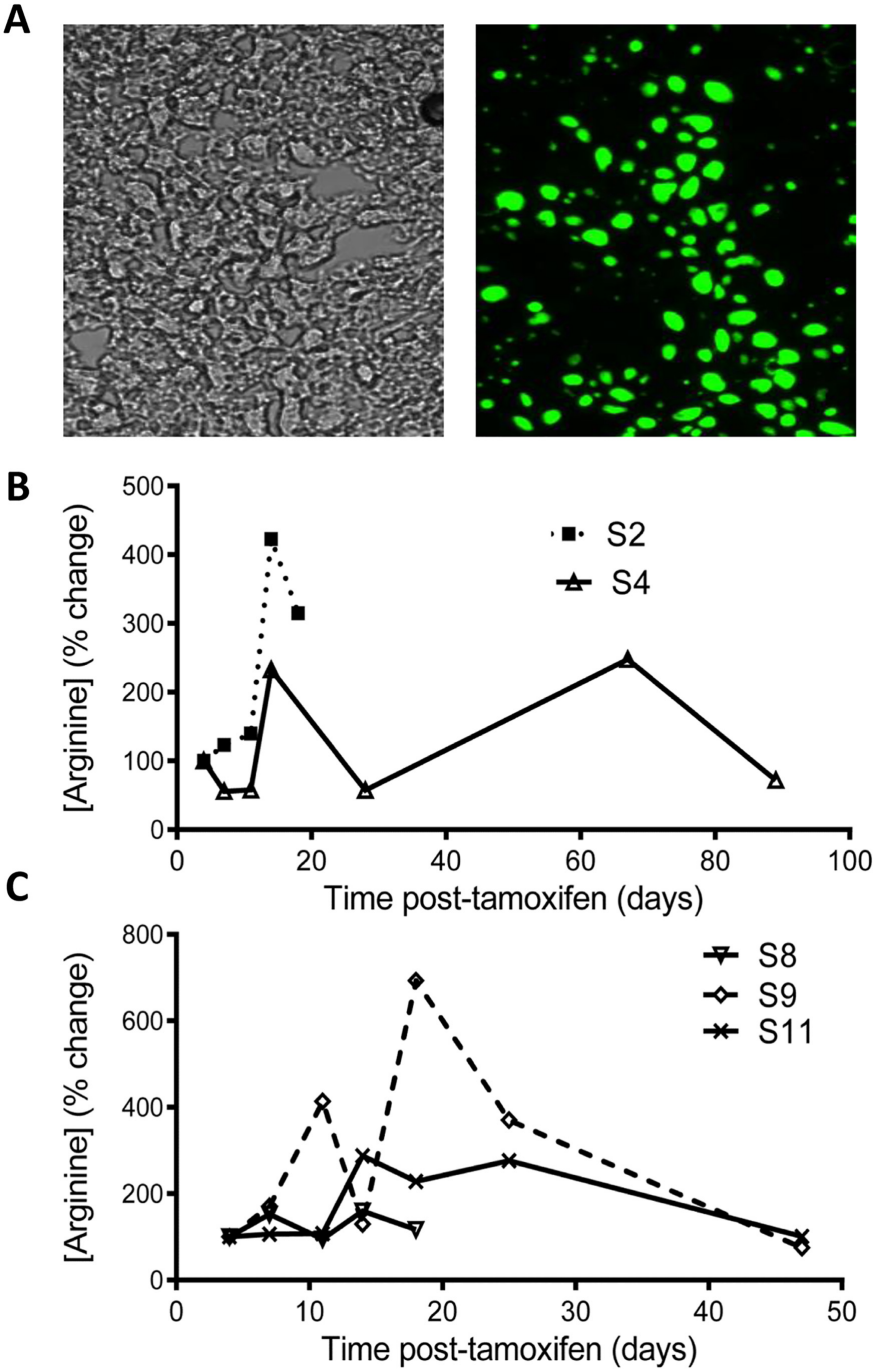
Transgenic expression of Arg1-eGFP rescues some male Arg1 knockout mice but variably corrects plasma arginine. (A) Phase contrast cryostat section (non-fixed, non-stained; left panel) and corresponding fluorescent image of liver section from mouse S9 (right panel) analyzed on Day +49 administered a single dose of 7.5x10^*10*^ gc AAVrh10.CB7.CI.Arg1-eGFP.WPRE.rBG 68 days earlier. Note widespread expression beyond boundaries of vessels. (B, C) Plasma arginine concentrations expressed as a percentage change. Samples taken at 4 days after the last tamoxifen injection in mice were set to 100%. Data are shown for two mice from Experiment 6 (Table 1; mouse S2 with low Arg1 enzyme activity and rescued mouse S4 with high Arg1 activity; see Fig 8D) and three mice from Experiment 7 (Table 1; non-rescued mouse S8 with high Arg1 activity, rescued mouse S9 with high Arg1 activity, and rescued mouse S11 with moderate Arg1 activity; see Fig 8D) are shown.

Based on the previous “rescue” of the juvenile lethal model of arginase-1 deficiency [13] with an rh10 AAV vector using a promoter that drives strong expression in neonatal-injected mice [18], we constructed a similar vector but encoding an Arg1-eGFP fusion (Fig 7A). We tested AAVrh10.CB7.CI.Arg1-eGFP.WPRE.rBG viral delivery by i.p. injection to tamoxifen-treated floxed Arg1-Cre mice (n = 34; 24 male/12 female), varying two parameters: (i) the viral dose (2.5x10^10^, 5x10^10^, 7.5x10^10^, 1.5x10^11^, 2x10^11^ gc/mouse); and (ii) the timing of viral delivery relative to tamoxifen-induced Arg1 deletion (Day -31 to Day -3; Table 1). Male mice fared better than females and 7 males (29%) were rescued with lifespan greatly extended, if the viral dose was 7.5x10^10^ gc or greater (Table 1; Fig 8A). In fact, there was evidence of significant expression of the Arg1-eGFP fusion by direct liver visualization under UV light (Fig 8B), by Western blot analysis (Fig 8C) and by measurement of enzyme activity (Fig 8D) in the livers of rescued male mice. The enzyme activity in the rescued mice ranged between 40–85% of normal wild-type levels. Upon section analysis of livers, highly expressing fluorescent cells were observed throughout this organ, not just immediately adjacent to vessels (Fig 9A). Plasma arginine, which usually increases 4- to 8-fold at humane endpoint in tamoxifen-treated inducible knockout Arg1 deficient mice [15], showed variable increases in the AAV-treated mice after tamoxifen treatment and no strong correlating trends with Arg1 enzyme activity (Fig 9B and 9C).

## Discussion

We report here the first attempts to rescue the lethal consequences in an inducible arginase-1 deficiency mouse model [10] using multiple strategies. The genetic disorder in mice, both the juvenile disorder [7] and the induced deficiency in mature animals [10,11], is much more severe than in humans perhaps due to an inability to induce compensatory arginase-2 expression. In our standard induced knockout model [10], mice progressively lose weight and require humane euthanization, regardless of age, about 2 weeks after the last tamoxifen dose with only ≈1.5% residual Arg1 protein (Fig 1; <10% enzyme activity based on a rather insensitive indirect enzyme assay). Previously, we speculated on the mechanisms for death in the model, which included hyperargininemia and decreases in plasma levels of several other amino acids, along with end-stage hyperammonemia [10]. Ornithine supplementation, a low-protein diet and administration of the ammonia-scavenging drug sodium phenylbutyrate, all clinically used therapies for arginase deficiency, were largely ineffectual at extending lifespan in the induced knockout mice for reasons that are not clear but might relate to some of the factors previously discussed in the creation of the model [10].

A transgenic approach to concomitantly induce expression of Arg1 throughout the body of mice using the ubiquitously expressed *Rosa26* genetic locus [20], while knocking out endogenous Arg1 did not prove to be effective in 4 week old mice but was modestly effective in neonatal mice (Figs 4–6). The reasons for lack of rescue may relate to two factors. The first is that expression in the liver, the site of most abundant Arg1 expression in the body of mice under normal circumstances, from the transgene was much less than anticipated. Although Arg1 mRNA in the transgenic mouse liver was greater than in the knockout mice not expressing the transgene, expression was only about 1% of endogenous levels. On the other hand, much higher Arg1 expression was observed in the brain and kidney of transgenic mice indicating differential tissue-specific expression from the *R26* locus. Other transgenic rescue strategies are being considered for future experiments. The second factor that may have limited this approach relates to the time course of Cre-based excision of the two cassettes (one which deletes exon 7 and 8 in *Arg1* to generate the knockout and the other which removes the PGK-Neo cassette to induce efficient transcription and downstream translation of the *Arg1* transgene). Perhaps the deleterious consequences of Arg1 knockout supersede/precede the induction of new Arg1 synthesis from the transgene so that, by the time there is sufficient expression, irreversible pathological sequelae ensue. Unfortunately, in this model we cannot distinguish between native Arg1 and transgenic-expressed Arg1.

Gene therapeutic approaches have been used previously in the juvenile lethal Arg1 disorder [8,12–15]. Adenoviral delivered Arg1 [12] or myocyte-directed adeno-associated viral (AAV) delivery of Arg1 [15] was largely ineffectual at rescuing the lethal phenotype. However, AAV-based expression via a beta-actin promoter driven Arg1 construct was much more successful, with long-term survival of mice that display only minimal neurological deficits but still possess some lingering biochemical defects [13,14]. Recent studies have indicated that these rescued adult mice, treated neonatally with AAV-Arg1, retain only 3% capacity of normal ureagenesis [21], which is quite striking. Our studies here indicate that it is impossible for induced Arg1 deficient mice to survive with less than 10% Arg1 protein/activity based on numerous Western blot analyses and enzyme activity assays on terminally ill mice with or without attempts at transgene delivery. In fact, we often achieved levels of Arg1 fusion protein expression via AAV in our early experiments using liver-directed delivery (TBG promoter and AAV8 serotype) in the 2–3% range of normal endogenous Arg1 liver expression (Fig 7). However, lifespan was only extended by a few days at most. We chose to use a fluorescent version of Arg1 (eGFP fused to C-terminus of enzyme) to distinguish it from endogenous murine Arg1 and to allow easy tracking of the enzyme. Based on the crystal structure of homotrimeric Arg1 [22] and the well-known independent folding of GFP, we reasoned that the fusion protein should be fully functional and tests using transfected cells confirmed that the fusion protein did indeed retain near full catalytic activity. However, for future therapeutic purposes it would be necessary to remove the fluorescent tag.

Since numerous tests with mice of various ages using the AAV8 viral construct, along with modifying viral dose and timing of administration of the viral vector were unsuccessful, we switched to an AAV vector serotype (rh10) with a potent promoter (hybrid CMV-chicken beta-actin) [19] that successfully rescued the juvenile lethal model of arginase-1 deficiency [13,14] but with the exception that here we used an Arg1-eGFP fusion. Interestingly, a proportion of male mice (around 30%) could be rescued using this vector, but not females. This may relate to the findings of others that AAV can more efficiently transduce liver cells of male mice compared to females [23] via an androgen-dependent mechanism. We are uncertain as to why only 1 of every 3 male mice was rescued. There was substantial heterogeneity of Arg1-eGFP 66 kDa protein expression in the liver of the AAV-treated “rescue” mice. However, they all displayed enzyme activity >40% of normal wildtype Arg1 activity with only minimal signs of endogenous liver Arg1 protein remaining after tamoxifen treatment (Fig 8). Moreover, some mice (S8, S10, S12) that had substantially restored liver Arg1 enzyme activity were not rescued (maximum 5 days lifespan extension), even though plasma arginine was well controlled (e.g. mouse S8, Fig 9C). Variable extra-hepatic expression of the Arg1-eGFP transgene and/or the lack of its ability to manage plasma arginine effectively (e.g. see variability for mice in Fig 9B and 9C) might explain the survival differences in male mice and why the majority of mice still die after induced Arg1 knockout by tamoxifen. The inducible Arg1 knockout, therefore, appears to be much more difficult to rescue than the juvenile lethal model by gene therapy methods. Future experiments should hopefully resolve these issues.
